# Requirement of HDAC6 for activation of Notch1 by TGF-β1

**DOI:** 10.1038/srep31086

**Published:** 2016-08-08

**Authors:** Brian Deskin, Joseph Lasky, Yan Zhuang, Bin Shan

**Affiliations:** 1 Tulane University Health Sciences Center, Department of Medicine, New Orleans, LA, 70119, USA; 2 Washington State University-Spokane, Elson S. Floyd College of Medicine, Department of Biomedical Sciences, Spokane, WA, 99210, USA

## Abstract

TGF-β1 is enriched in the tumor microenvironment and acts as a key inducer of epithelial to mesenchymal transition (EMT) in lung cancer. The NOTCH signaling pathway is conserved across species and is an essential pathway for development, cell differentiation, and cancer biology. Dysregulation of Notch signaling is a common feature of non-small cell lung cancer (NSCLC) and is correlated with poor prognosis. Crosstalk exists between the NOTCH and TGF-β signaling pathways in EMT. Herein we report that histone deacetylase 6 (HDAC6) modulates TGF-β1-mediated activation of the Notch pathway. HDAC6, a primarily cytoplasmic deacetylase, mediates TGF-β1-induced EMT in human lung cancer cells. Inhibition of HDAC6 with a small molecule inhibitor, namely tubacin or with siRNA attenuated TGF-β1-induced Notch-1 signaling. We show that TGFβ-1-induced EMT is accompanied by rapid HDAC6-dependent deacetylation of heat shock protein 90 (HSP90). Consistently, inhibition of HSP90 with its small molecule inhibitor 17AAG attenuated expression of TGF-β1-induced Notch-1 target genes, HEY-1 and HES-1. These findings reveal a novel function of HDAC6 in EMT via mediating the TGF-β-Notch signaling cascade, and support HDAC6 as a key regulator of TGFβ-induced EMT in NSCLC. This work suggests that HDAC6 may be an attractive therapeutic target against tumor progression and metastasis.

Epithelial-mesenchymal transition (EMT) is a process in which epithelial cells lose their characteristic cell-cell junctions and polarization of cell-surface molecules while acquiring properties typical of mesenchymal cells, which leads to metastasis to distant sites^1^. Transforming growth factor (TGF)-β1 is a well-established inducer of EMT in cancer^2^. Notch signaling contributes to EMT in cancer cells by suppressing expression of E-cadherin and thereby mediates invasion and metastasis^3^,^4^. Canonical Notch signaling requires cell-cell contact between Notch receptor presenting cells with cells presenting one of its cognate ligand family members. This interaction results in two proteolytic events, first an extracellular cleavage event dependent on ADAM metalloproteases followed by an intracellular cleavage event dependent on the γ-secretase complex. This final cleavage of the Notch receptor releases the intracellular domain (Notch ICN) and allows for translocation of Notch ICN to the nucleus where it interacts with RBP-J (Recombinant Signal Binding Protein) and additional coactivators to initiate transcription of Notch target genes^5^. Non-canonical activation of Notch signaling can occur in a ligand-dependent or independent fashion and Notch ICN can signal independent of RBP-J^4^,^6^,^7^. TGFβ-induced EMT can be attenuated with pharmacological inhibition of Notch or by genetic knockdown of Notch target gene HEY-1 or Notch ligand Jagged-1^8^. Understanding the pathological pathways that crosstalk with Notch will aid in devising therapeutic strategies to target EMT. Emerging evidence documents that crosstalk between TGFβ and Notch signaling promotes EMT^8^,^9^.

Lysine acetylation is a critical post-translational modification in regulation of protein function and is a key component of many signaling networks. An acetyl group can be added to a lysine residue by histone acetyl transferases (HATs) and removed by histone deacetylases (HDACs). HDAC6 is unique amongst its family members in that it has dual deacetylase domains, an ubiquitin binding motif, and primarily resides in the cytoplasm though under certain contexts HDAC6 can shuttle between the nucleus and cytoplasm^10^,^11^,^12^. A well characterized substrate of HDAC6 is heat shock protein 90 (HSP90); HDAC6-mediated deacetylation of key lysine residues of HSP90 is required for proper maturation of receptors like the glucocorticoid receptor and ErbB2^13^,^14^.

The current study investigated a role of HDAC6 in TGF-β1-induced Notch signaling. We show that TGF-β1 induces HDAC6-dependent deacetylation of HSP90 in A549 cells, which occurs concurrently with activation of Notch signaling. Pharmacological inhibition of HDAC6 attenuates TGF-β-induced activation of Notch signaling and expression of its target genes HEY-1 and HES-1 in both A549 and H1299 cells. In addition, we demonstrate that there is direct interaction between HSP90 and the Notch-1 receptor, and abrogation of TGFβ-induced Notch target genes HEY-1 and HES-1 by inhibition of HSP90.

## Results

### TGF-β1-induces activation of Notch1 ICN cleavage and Notch effector gene expression in A549 cells

**Figure 1:**
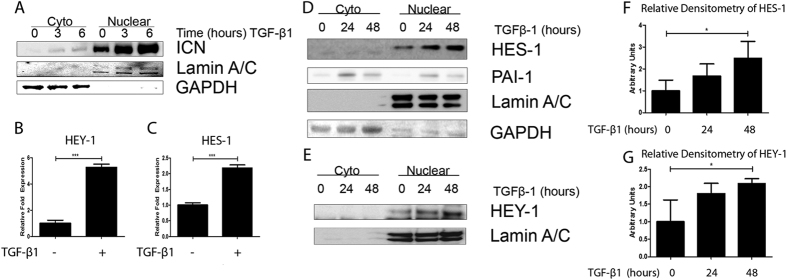
TGF-β1-induced activation of Notch1 ICN Cleavage in A549 cells. (**A**) Western analysis of cytoplasmic and nuclear fractions demonstrating nuclear translocation Notch1 ICN after treatment with TGFβ-1. (**B,C**) qPCR analysis of Notch signaling genes HEY-1 (**B**) and HES-1 (**C**) in A549 cells treated with 2.5 ng/mL TGFβ-1 for 24 hours. The –fold change of each transcript was obtained by setting the value of the unexposed cells to 1. (**D**,**E**) Western analysis of Notch signaling genes HES-1 (**D**) and TGF-β1 responsive gene PAI-1, and HEY-1 (**E**) in A549 cells two separate blots of the same experiment treated with 2.5 ng/mL TGFβ-1 for a time course over 48 hours. (**F**,**G**) Densitometry analysis of three independent experiments and probed for HES-1 (**F**) and HEY-1 (**G**). Data for qPCR presented as mean +/− SEM of triplicate wells and are representative of three independent experiments statistically analyzed using one-way ANOVA. Data for densitometry analysis presented as +/− STD of three independent experiments statistically analyzed using unpaired T-tests. *P < 0.05, **P < 0.01 and ***P < 0.001 compared with the relative control.

To establish the sequence of events for Notch signaling activation by TGF-β1, we carried out a series of time course experiments examining cleavage of the Notch1 Receptor and nuclear translocation to activate downstream effector gene expression in A549 cells. Accumulation of nuclear intracellular Notch1 domain (ICN) was detected by fractionation of cell lysate followed by western blot analysis. An increase in nuclear Notch ICN is evident within three hours of treatment with TGF-β1 (2.5 ng/ml), with peak nuclear translocation occurring at six hours after TGF-β1 stimulation (Fig. 1A). Equal loading of cytoplasmic and nuclear extract among samples was confirmed by probing for GAPDH and lamin A/C, respectively. As shown in Fig. 1B,C, significant increases in transcripts of Notch1 downstream effector genes HEY-1 and HES-1 were detected as measured using qPCR after 24 hours exposure to TGF-β1. Western blot analysis was also performed on fractionated cell extracts from A549 cells treated with TGF-β1 for 24 and 48 hours and densitometry analysis performed (Fig. 1F,G). Notch effector genes HEY-1 and HES-1 showed a significant increase in nuclear accumulation over 48 hours of TGF-β1 stimulation. Activation of the canonical TGFβ pathway was confirmed by an increase in the protein levels of PAI-1 in the TGF-β1-treated cells (Fig. 1D). These results validate A549 as an experimental system to study TGF-β-induced Notch signaling.

### Requirement of HDAC6 for TGF-β1-activation of Notch signaling in A549 cells

**Figure 2:**
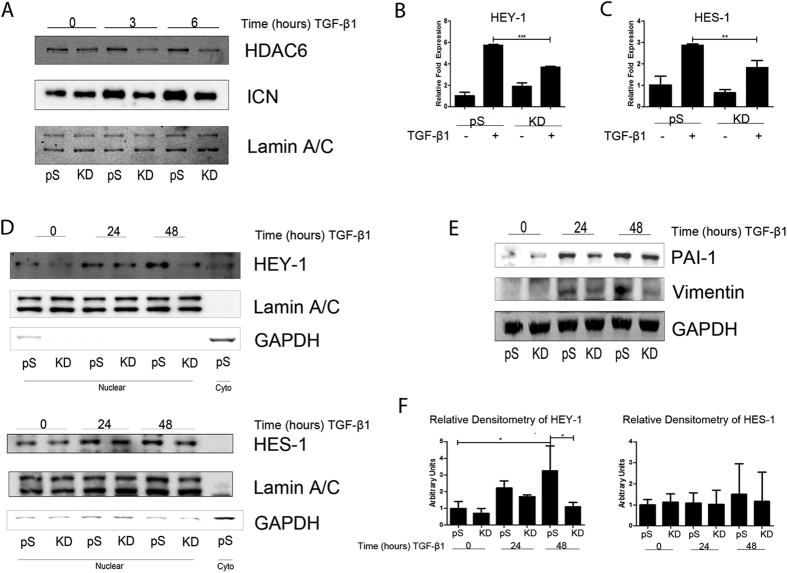
HDAC6 requirement for TGF-β1-activation of Notch Signaling in A549 cells. (**A**) Serum-starved A549 variants were exposed to TGF-β1 (2.5 ng/ml) for the indicated duration, cell lysates were fractionated to cytoplasmic and nuclear extracts and protein levels of nuclear ICN and HDAC6 were examined by Western analysis. (**B**) RNA was isolated from A549 variants exposed to TGF-β1 (2.5 ng/ml) for 24 hours. Quantitative RT-PCR was carried out for HEY-1. (**C**) Same experimental conditions as in B, except the transcript of interest examined was HES-1. The –fold change of each transcript was obtained by setting the value of the unexposed pS cells to 1 (**D**) Experimental conditions and fractionation of serum starved A549 variant cell lysates were the same as in A, except the duration of TGF-β1 treatment was for 24 and 48 hours. Protein levels of HEY-1 and HES-1 in the nuclear extract were examined by western analysis. (**E**) Cytoplasmic fraction of the same experiment as D, except TGF-β1 responsive genes PAI-1 and Vimentin protein levels were examined by western analysis. (**F**) Densitometry analysis of western blots from three independent experiments represented in panel (**D**); levels of HEY-1 and HES-1 were relativized to lamin A/C. For qPCR analysis data presented as mean +/− SEM of triplicate wells and are representative of three independent experiments statistically analyzed using one-way ANOVA. For densitometry analysis data presented as mean +/− STD of three independent experiments statistically analyzed using one-way ANOVA. *P <0.05, **P < 0.01, and ***P < 0.001 compared with the relative control.

**Figure 3:**
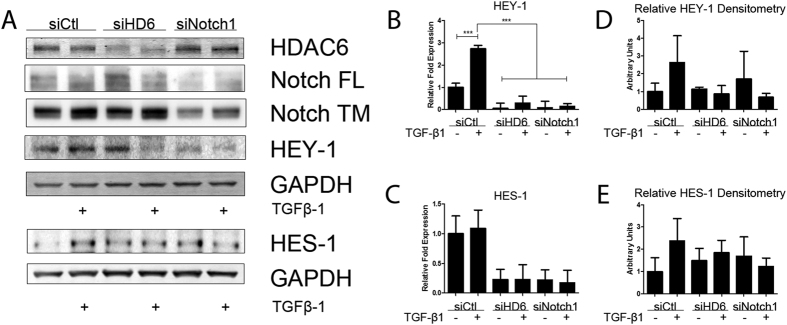
Genetic knockdown of HDAC6 reduces TGF-β1-activation of Notch Signaling in A549 cells. (**A**) A549 cells transiently transfected with siRNA targeting HDAC6, Notch1, or non-specific control were serum starved and treated with TGF-β1 for 24 hours. Whole cell lysate protein levels of HDAC6, Notch1, HEY-1, and HES-1 were examined by western analysis (separate blots provided of the same experiment). (**B,C**) A549 cells treated as in A, except RNA was isolated and quantitative RT-PCR was carried out for HEY-1 (**B**) and HES-1 (**C**). (**D**,**E**) Densitometry analysis of protein levels of HEY-1 (**D**) and HES-1 (**E**) from three independent experiments. For qPCR analysis data presented as mean +/− SEM of triplicate wells and are representative of three independent experiments statistically analyzed using one-way ANOVA. *P < 0.05, **P < 0.01 and ***P < 0.001 compared with the relative control.

We hypothesized that HDAC6 is required for TGFβ-induced Notch signaling because our earlier work demonstrated an HDAC6 requirement for TGFβ-induced EMT^15^. Therefore, we examined the sequence of signaling events from Notch ICN cleavage to downstream gene expression to identify where HDAC6 intercepts. To test whether TGF-β-induced Notch signaling is dependent on HDAC6 we employed a model of A549 variants expressing HDAC6 targeting siRNA (HDAC6-knockdown (KD) cells) and compared these variants to the empty vector (pS) variants and examined nuclear fractions of cell lysates. After stimulation with TGF-β1, cleavage of full-length Notch in pS cells displayed a similar time course as the parental A549 cell line with nuclear accumulation of Notch ICN evident at 3 hours (pS = 2.73 +/− 0.54 compared to KD = 2.05 +/− 0.17) and peaking at 6 hours (pS = 2.91 +/− 0.25 compared to KD = 1.47 +/− 0.73) compared to KD cells (Fig. 2A). We examined the expression of Notch activated genes HEY-1 and HES-1 in response to TGF-β1 stimulation (2.5 ng/ml) using quantitative RT-PCR. Compared to KD cells, pS cells showed a greater increase in response to TGF-β1 in transcript levels of HEY-1 and HES-1 (Fig. 2B,C). Western blot analysis of the nuclear fraction confirmed greater expression levels of HEY-1 and HES-1 in pS cells that were stimulated with TGF-β1 (2.5 ng/ml) compared to KD cells over a 48-hour treatment period (Fig. 2D). To confirm concurrence of TGF-β1 induced EMT and NOTCH signaling we chose to analyze two EMT markers vimentin and PAI-1 in the cytoplasmic fraction and demonstrated alterations in the expression of these markers in response to TGF-β1 (Fig. 2E). As an alternative genetic approach we transiently transfected A549 cells with HDAC6-targeting siRNA oligos (25 pm), Notch-1-targeting siRNA oligos (25 pm), or nonspecific control siRNA oligos (25 pm). Transcript levels of HEY-1 were lower in the HDAC6-targeting siRNA group than the control siRNA group in response to TGF-β1 (Fig. 3B). Western blot analysis of whole cell lysate revealed only the HDAC6-targeting siRNA group exhibited an attenuated increase in the protein levels of HEY-1 and HES-1 in response to TGF-β1 when compared to the control siRNA group (Fig. 3A). Densitometry analysis of immunoblots from three independent experiments revealed no significant increase in HEY-1 protein levels, though a noticeable trend of HEY-1 and HES-1 induction is demonstrated in the control siRNA group exposed to TGF-β1 compared to the HDAC6-targeting siRNA group exposed to TGF-β1 (Fig. 3D,E).

**Figure 4:**
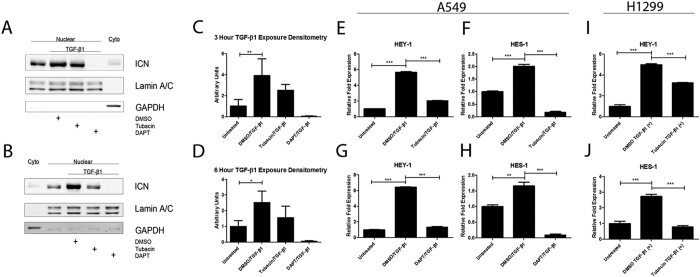
Pharmacological inhibition of HDAC6 abrogates TGF-β1-activation of Notch Signaling in A549 and H1299 cells. (**A**) Serum-starved A549 cells were pre-treated with either 8 μM tubacin, 10 μM DAPT, or an equivalent volume of DMSO for six hours before being exposed to TGF-β1 (2.5 ng/ml) for three hours. Cell lysates were fractionated to cytoplasmic and nuclear extracts and protein levels of nuclear ICN and were examined by western analysis. (**B**) Same experimental design as panel (**A**) except the duration of TGF-β1 exposure was for 6 hours. (**C**,**D**) Densitometry analysis of western blots from three independent experiments represented in panels (**A**,**B**), respectively; levels of nuclear cleaved Notch1 (ICN) were relativized to lamin A/C. (**E**,**F**) Serum-starved A549 cells were pre-treated with 8 μM tubacin for six hours before being exposed to TGF-β1 (2.5 ng/ml) for 24 hours. RNA was isolated and quantitative RT-PCR was carried out for HEY-1 (**E**) and HES-1 (**F**). (**G**,**H**) Serum-starved A549 cells were pre-treated with 10 μM DAPT for six hours before being exposed to TGF-β1 (2.5 ng/ml) for 24 hours. RNA was isolated and quantitative RT-PCR was carried out for HEY-1 (**G**) and HES-1 (**H**). (**I**,**J**) Serum-starved H1299 cells were pre-treated with 8 μM tubacin for six hours before being exposed to TGF-β1 (10 ng/ml) for 24 hours. RNA was isolated and quantitative RT-PCR was carried out for HEY-1 (**I**) and HES-1 (**J**). The –fold change of each transcript was obtained by setting the value of the untreated cells to 1. Data for qPCR analysis presented as mean +/− SEM of triplicate wells and are representative of three independent experiments; data for densitometry analysis presented as mean +/− STD and are representative of three separate experiments statistically analyzed using one-way ANOVA. *P < 0.05, **P < 0.01 and ***P < 0.001 compared with the relative control.

As an alternative strategy to inhibit HDAC6 activity we employed an HDAC6-specific inhibitor, tubacin (8 μm). We chose to probe early events in TGF-β-induced Notch signaling in A549 cells. Tubacin treatment inhibited nuclear accumulation of cleaved Notch-1 at 3 and 6 hours after exposure to TGF-β1 (Fig. 4A,B). Cells treated with TGF-β1 for 24 hours in the presence of tubacin displayed significantly reduced expression of the Notch activated genes HEY-1 and HES-1 in A549 cells (Fig. 4E,F). As a set of control experiments we treated cells with the γ-secretase inhibitor DAPT (10 μm) and stimulated them with TGF-β1 for 24 hours to confirm that blockade of NOTCH activation by the γ-secretase inhibitor DAPT inhibited expression of HEY-1 and HES-1 (Fig. 4G,H). Densitometry analysis of immunoblots from three independent experiments revealed significant changes in only the TGF-β1-treated DMSO controls compared to the untreated controls for both the 3- and 6-hour time points (Fig. 4C,D). To test whether inhibition of HDAC6 in TGF-β-induced Notch signaling extends to other cell culture models of NSCLC we employed H1299 cells. Similar to A549 cells, tubacin treatment at 8 μm significantly abrogated induction of HEY-1 and HES-1 in response to TGF-β1 in H1299 cells (Fig. 4I,J).

### TGF-β1-induced deacetylation of HSP90 by HDAC6 is required for Notch1 activation in A549 cells

**Figure 5:**
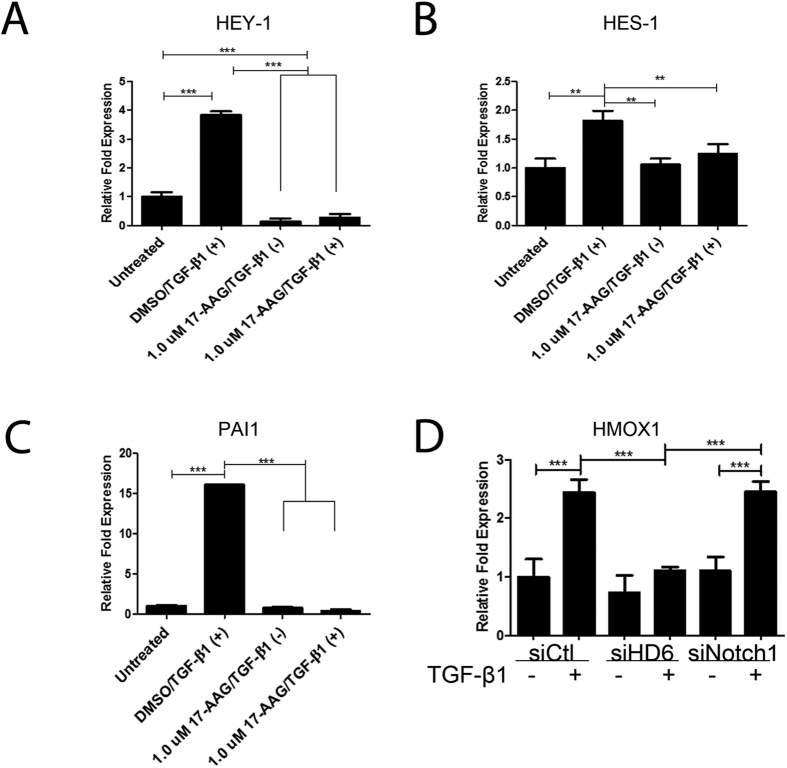
HSP90 inhibition effect on TGF-β1-induced Notch signaling in A549 cells. (**A**–**C**) Serum-starved A549 cells were pre-treated for six hours with either 1 μM or 10 μM 17-AAG or an equivalent volume of DMSO for six hours before being exposed to TGF-β1 (2.5 ng/ml) for 24 hours. RNA was isolated and quantitative RT-PCR was carried out for HEY-1, HES-1, and PAI1 transcripts. (**D**) A549 cells transiently transfected with siRNA targeting HDAC6, Notch1, or non-specific control were serum starved and treated with TGF-β1 for 24 hours. RNA was isolated and Quantitative RT-PCR was carried out for HMOX1 transcripts. The –fold change of each transcript was obtained by setting the value of the untreated cells to 1. Data presented as mean +/− SEM of triplicate wells and are representative of three independent experiments (**A**–**C**) and D as mean +/− STD of triplicate wells from a single independent experiment statistically analyzed using one-way ANOVA. *P < 0.05, **P < 0.01 and ***P < 0.001 compared with the relative control.

We questioned whether HDAC6 mediated Notch-1 activation by TGF-β1 via deacetylation of its substrate/s. We chose to test whether inhibition of HSP90 with the geldanamycin analog, 17-AAG (1 μm), would inhibit TGF-β-induced Notch signaling because HSP90 is a well-documented substrate of HDAC6 deacetylase activity and involved in the TGF-β1 signaling. As an initial screen we stimulated A549 cells with TGF-β1 for 24 hours with or without exposure to 17-AAG. We probed for changes in mRNA levels of Notch activated genes HEY-1 and HES-1 and the TGF-β activated gene PAI-1. Inhibition of HSP90 resulted in abrogation of TGF-β-induced expression of HEY-1, HES-1, and PAI1 mRNA levels as measured by quantitative RT-PCR (Fig. 5A–C). We also analyzed mRNA isolated from transient siRNA transfections against HDAC6 or Notch1 to measure the transcript levels of heme oxygenase-1 (HMOX1) that served as an indicator of the p38 pathway activated by HSP90 upon exposure to TGF-β^16^,^17^. The HDAC6-specific siRNA, but not the NOTCH1-specific siRNA, abrogated expression of HMOX1 in response to TGF-β1 stimulation (Fig. 5D).

**Figure 6:**
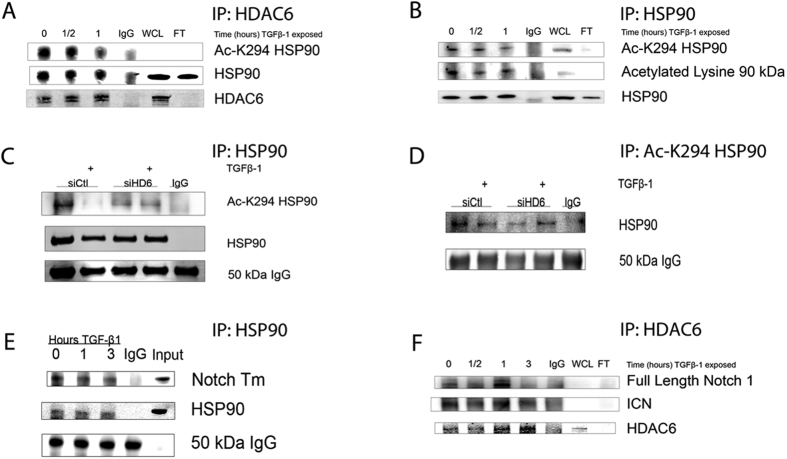
TGF-β1-induced deacetylation of HSP90 by HDAC6 in A549 cells. (**A**,**B**,**E**,**F**) Immunoprecipitation of respective target antigens from whole cell lysates of serum-starved A549 cells treated with TGF-β1 (2.5 ng/ml) for the indicated time and examined by western analysis. (**A**) Western analysis for acetylated-K294 HSP90 co-precipitated with anti-HDAC6. (**B**) Western analysis of acetylated-K294 HSP90 immunoprecipitated with anti-HSP90. (**C**,**D**) Serum-starved A549 cells transiently transfected with siRNA targeting HDAC6, or non-specific siRNA treated with TGF-β1 for one hour. Cell lysates were immunoprecipitated with anti-HSP90 (**C**) or anti-Ac-K294 HSP90 (**D**) and western analysis of Ac-K294 HSP90 (**C**) or total HSP90 (**D**). (**E**,**F**) Immunoprecipitation of HDAC6 and HSP90, respectively, of same cell lysates as in (**A**,**B**) and examined by western analysis for co-precipitated full length Notch1 or transmembrane Notch1 (Notch Tm). WCL = whole cell lysate; FT = flow through.

We hypothesized that when HDAC6 is activated by TGFβ1 stimulation, it will also deacetylate its substrates, specifically HSP90^16^. To test this hypothesis we employed a series of immunoprecipitation reactions. Pulldown of endogenous HDAC6 revealed that HSP90 co-immunoprecipitated with HDAC6 and displayed a time-dependent decrease in HDAC6-bound HSP90 with lysine 294 acetylated, an established HDAC6 target site, as revealed by an acetylation site-specific K294-HSP90 antibody (Fig. 6A). Deacetylation occurred at thirty minutes (0.68 +/− 0.07) and peaked at one hour after treatment with TGF-β1 (0.49 +/− 0.08). The same lysates were used for a reciprocal pulldown of HSP90 followed by immunoblot analysis of the precipitates for acetyl-K294 HSP90 (Ac-K294-HSP90) as well as total acetylation. Consistently, treatment with TGF-β1 induced deacetylation of HSP90 at lysine 294 at 30 minutes, and peaked at one hour (Fig. 6B). These results led us to hypothesize that HSP90 deacetylation by HDAC6 after TGF-β1 treatment is a signaling feature that correlates with cleavage of the Notch-1 receptor. To test whether HDAC6-dependant deacetylation of HSP90 is linked to Notch-1 cleavage we performed co-immunoprecipitation to examine reactions of endogenous HSP90 and Notch-1 ICN. Immunoblot analysis of the pulldown assays demonstrate that HSP90 co-immunoprecipitated with Notch-1 (Fig. 6E). Similarly, pull down of HDAC6 demonstrated that both the full length Notch-1 receptor and the cleavage product, Notch-1 ICN, co-immunoprecipitated with HDAC6 (Fig. 6F). To determine the role of HDAC6 in deacetylation of HSP90, we transiently transfected A549 cells with HDAC6-specific siRNA oligos or with nonspecific siRNA oligos and analyzed HSP90 acetylation status in response to TGFβ-1 stimulation. Western analysis of HSP90 pulldown assays demonstrated that after one hour of TGF-β1 stimulation lysine 294 of HSP90 was deacetylated in the non-specific siRNA group while this deacetylation event was not evident in the HDAC6-specific siRNA group. Reciprocal pulldown of Ac-K294-HSP90 further confirmed a TGF-β1 induced deacetylation of lysine 294 of HSP90 only in the nonspecific siRNA group (Fig. 6C,D).

## Discussion

EMT is an established feature of tumorigenesis. This study investigates the mechanisms that mediate TGF-β1-induced EMT. HDAC6 is required for cleavage of Notch-1 ICN and for maximal expression of Notch target genes activated by TGF-β1. Blockade of Notch by siRNA directed against HDAC6 or Notch-1 attenuates expression of EMT genes HEY-1 and HES-1 (Fig. 3). In both A549 and H1299 cells pharmacological inhibition of HDAC6 with tubacin abrogates induction of TGF-β1-induced expression of HEY-1 and HES-1 (Fig. 4). TGF-β1 induces HDAC6-dependent deacetylation of HSP90 that correlates with activation of Notch-1 signaling suggesting that deacetylation of HSP90 by HDAC6 may be involved for Notch-1 cleavage. These results establish HDAC6 as a key therapeutic target against EMT in cancer.

Overexpression of HDAC6 is a common feature in lung adenocarcinoma cell lines and negatively correlates with prognosis of lung adenocarcinoma patients^18^. HDAC6 overexpression in adenocarcinoma cells promotes proliferation through its deacetylase activity. Inhibition of HDAC6 in lung adenocarcinoma cell lines induces apoptosis by conferring sensitivity to gefitinib, an epidermal growth factor (EGFR) inhibitor^18^. Overexpression of HDAC6 confers resistance to the multi-kinase inhibitor, sorafenib in NSCLC lines. Sorafenib enhances expression of HDAC6 and activates the EGFR pathway. In NSCLC HDAC6 inhibition synergizes with sorafenib to inhibit proliferation and enhance apoptosis^19^.

Identification of HDAC6 as a TGF-β1 induced deacetylase for deacetylation of HSP90 illustrates a novel mechanism that regulates EMT. Our study suggests an HDAC6–dependent deacetylation mechanism of HSP90 in human lung adenocarcinoma cell line A549 as a feature of TGF-β1 stimulation. Further, deacetylation of HSP90 is coupled with Notch-1 activation regulated by HDAC6 (Fig. 6). More interestingly, deacetylation of HSP90 correlates with cleavage of Notch-1 ICN and nuclear translocation. Given that aberrant activation of notch signaling is a common feature of EMT and contributes to non-small cell lung cancer through maintenance of the cancer stem cell compartment it is conceivable that TGF-β1-induced HDAC6-dependent deacetylation of HSP90 contributes to key signaling networks implicated in EMT^20^. Activation of Notch1 induces unchecked expansion of type II alveolar epithelial cells in a transgenic mouse model of alveolar-specific expression of activated Notch. Crossing these mice with a mouse line of conditionally overexpressed MYC in the alveolar epithelium results in lung adenocarcinomas^21^. We hypothesized that HDAC6 activation is an event that occurs in response to TGFβ-1 and can stimulate HDAC6-dependant deacetylation of HSP90. Specifically, a putative site of HSP90 deacetylation, lysine 294, that regulates HSP90 chaperone function^22^. These findings suggest that HDAC6-dependent regulation of HSP90 is an integral component of TGF-β1-induced EMT.

The importance of EMT in tumor progression and fibrosis has driven the field of signal transduction and aided in identifying therapeutic targets. The present study defines a novel role of HDAC6 in the TGF-β1-induced Notch signaling pathway. Non-canonical activation of the Notch pathway by TGF-β1 requires intact HDAC6 expression and function (Figs 2, 3, 4). Notch signaling contributes to NSCLC by helping to maintain the cancer stem cell population^23^. Our results suggest a critical role of interactions among HDAC6, HSP90, and Notch1 in TGF-β1-induced Notch signaling (Figs 5 and 6). Supporting evidence of a chaperone complex involved in the Notch pathway is provided by a very recent report that documents an HSP70/Notch-1 interaction required for nuclear transport in CD4 T cells^24^. It is reasonable to speculate that HSP90 and HSP70 could both function to stabilize the Notch receptor and aid in context-dependent signaling events.

The current study demonstrates HDAC6 is required for activation of Notch-1 signaling in response to TGF-β1 in human lung adenocarcinoma cells. Our results also show interplay amongst HDAC6, HSP90, and Notch-1 during TGFβ-1-induced EMT. These findings reveal a novel function for HDAC6 as a regulator of EMT through modulation of Notch signaling and warrant further investigation of HDAC6-dependent signaling pathways in NSCLC.

## Materials and Methods

### Cell culture and treatments

Human lung adenocarcinoma cell lines A549 and NCI-H1299 were purchased from the ATCC biological resource center (Manassas, VA). All cells were cultured in Dulbecco’s modified Eagle’s Medium (Gibco) containing 10% fetal bovine serum (v/v), and 100 μg/ml penicillin and 100 μg/ml streptomycin at 37 °C with atmospheric conditions of 95% air and 5% CO_2_. Recombinant human TGF-β1 was purchased from R&D systems (Minneapolis, MN). In the selected experiments, A549 cells were cultured in low serum DMEM containing 0.5% FBS overnight prior to exposure to TGF-β1; exposure to either the HDAC6-specific inhibitor, tubacin at 8 μM concentrations, the γ-secretase inhibitor, DAPT, at 10 μM concentrations, or the HSP90 inhibitor, 17AAG, at 1 μM concentrations for 6 hours followed by treatment with TGF-β1 (2.5 ng/mL). A549 variants with intact endogenous expression of HDAC6 (pS) or decreased HDAC6 expression by RNA interference (KD) were developed using retroviral siRNA expression vectors and provided in kind by Tso-pang Yao of Duke University^25^.

### Reagents and antibodies

Small molecules tubacin, an HDAC6-specific inhibitor, and DAPT, a γ-secretase-specific inhibitor were both purchased from Sigma (St. Louis, MO, USA). Small molecule 17AAG was purchased from Cell Signaling Technology (Danvers, MA, USA). siRNA targeting HDAC6 (SI00084000, SI00084014, SI02663808, SI02757769) Notch1 (SI00119035), and AllStars negative control siRNA (SI03650318) were purchased from Qiagen (Valencia, CA, USA).

The following rabbit monoclonal antibodies were used for immunoblots: Anti-Notch 1 (D6F11), anti-cleaved Notch 1-ICN (D3B8), and anti-GAPDH (14C10) were purchased from Cell Signaling Technologies (Danvers, MA, USA) and used for western analysis at 1:1,000, 1:1,000, and 1:3,000 respectively. Mouse monoclonal antibody for Lamin A/C (4C11) was purchased from Cell Signaling Technologies was used at 1:2,000. Rabbit polyclonal anti-HDAC6 (H300) was purchased from Santa Cruz (Dallas, TX, USA) and was used for western analysis at 1:1,000. Rabbit polyclonal antibodies HEY1 (ab22614) and HES1 (ab49170) were purchased from Abcam (Cambridge, MA, USA) and used for western analysis both at 1:500. Anti-PAI1 rabbit polyclonal antibody (500-P260) was purchased from PeproTech (Rocky Hill, NJ, USA) and was used for western analysis at 1:2,000. Mouse monoclonal anti-vimentin (clone V9) was purchased from Sigma-Aldrich (St. Louis, MO, USA) and used for western analysis at 1:1,000. Anti-HSP90 monoclonal mouse ascites fluid (clone D7α) was provided by Dr. Charles Miller III of Tulane University who received it from Dr. Marc Cox of University of Texas, El Paso, and was used for western analysis at 1:3,000. Rabbit polyclonal anti-Acetyl-specific-K294-HSP90α (600-401-981) was purchased from Rockland Antibodies (Limerick, PA, USA) and was used for western analysis at 1:1,000. Rabbit polyclonal affinity-purified pan-specific anti-acetyl-Lysine was purchased from ImmuneChem Pharmaceuticals Inc. (Burnaby, British Colombia, Canada) and was used for western analysis at 1:1,000.

### Cell Fractionation and Immunoblot Analysis

Cytosolic and nuclear protein fractions were extracted from the treated cells using the NE-PER^®^ kit (Pierce). Electrophoretic grade reagents: MOPS SDS running buffer, NuPAGE Transfer Buffer, and NuPAGE precast gels were obtained from Invitrogen (Carlsbad, CA). Tween 20 was purchased from Fisher Scientific (Waltham, MA). Protease inhibitor cocktail tablets (Complete Tablets) were purchased from Roche (Nutley, NJ). Protease inhibitor PMSF was purchased from Sigma (St. Louis, MO). Protein concentrations were quantitated using the Bio-Rad DC^TM^ Protein Assay kit (Hercules, CA). Both cytoplasmic extract buffer and nuclear extract buffer contained protease inhibitory cocktail and 100 nM PMSF. Before lysis, cells were washed in PBS and trypsinized. Cells were collected and pelleted by centrifugation. Whole cell lysate was collected using RIPA containing PMSF and protease inhibitory cocktail. For fractionation of cytoplasmic and nuclear extracts, the manufacturer’s protocol was followed (Peirce). Equal amounts of protein from each sample were prepared by resuspension in NuPAGE LDS-sample buffer followed by heating to 70 ° for 5 minutes. Samples were resolved using precast 4–12% Bis-Tris NuPAGE gels (Invtirogen (Carlsbad, CA)) and transferred to a nitrocellulose membrane (Immobilon). Membranes were blocked for 1 hour in 5% non-fat dry milk in 1% TBS-T (100 mM Tris, pH 7.6, 0.9% NaCl, and 0.1% Tween 20). Membranes were incubated overnight with appropriate antibodies in 4 °C followed by washing four times with PBS-T. Appropriate secondary antibodies were then added and incubated for one hour at room temperature followed by washing four times with PBS-T.

### Immunoprecipitation

For Fig. 6A–D, lysates for immunoprecipitation were prepared in an IP extract buffer composed of 10% glycerol, 50 mM Tris, 150 mM NaCl, 0.5% IGEPAL, pH 7.7 in ddH_2_O containing 500 nm TSA (Sigma, St. Louis, MO, USA), protease inhibitory cocktail (Roche, Indianapolis, IN, USA) and 100 nM PMSF (Sigma, St. Louis, MO, USA). Cells were washed with PBS prior to lysis with IP buffer. Cell lysates were scraped on ice, passed through a 28 gauge needle five times and centrifuged at 16,000 rcf for 10 minutes. The supernatants were transferred to fresh tubes and protein concentrations were measured using the Bio-Rad DC^TM^ Protein Assay kit. For Fig. 6A–D, 4 μg of HDAC6 antibody or 1 μL of HSP90 ascites fluid were added to 500 μg of lysate and incubated overnight on a shaking tumbler overnight at 4 °C. 4 μg of IgG matching isotype were added to an equivalent volume of pooled lysates and incubated overnight on a shaking tumbler overnight at 4 °C. The following day 25 μL of pre-washed protein AG magnetic beads (Pierce, Appleton, WI, USA) were incubated with the lysate-immunecomplex for one hour at room temperature on a tumbler. Beads were washed three times with IP buffer. After the final wash, beads were collected an all residual wash buffer was removed, 100 μL of Laemli sample buffer was added to the beads and heated to 70 °C for ten minutes. Beads were collected and the samples were resolved by gel electrophoresis and analyzed by western analysis. For Fig. 6E,F cell lysates for immunoprecipitation were prepared in RIPA buffer containing 500 nM TSA, protease inhibitory cocktail, and 100 nM PMSF. The same protocol was then followed throughout the rest of the analysis, using 3 μL of HSP90 ascites fluid to one mg of lysate or 10 μg of anti-Acetyl-specific-K294-HSP90α to IP 500 ng of lysate, respectively.

### Quantitative Reverse Transcription PCR

Expression of mRNA levels of genes of interest in A549 was determined via quantitative RT-PCR, 36B4 was used as the control transcript for all datasets. The primers used are as follows: human HES1(NM_00524), forward primer (positions 479–501; 5′-GGAAATGACAGTGAAGCACCTCC-3′) and reverse primer (positions 587–608; 5′-GAAGCGGGTCACCTCGTTCATG-3′); human HEY1 (NM_001040708) forward primer (positions 271–295; 5′-AGAGTGCGGACGAGAATGGAAACT-3′) and reverse primer (positions 427–451; 5′-ACCAGCCTTCTCAGCTCAGACAAA-3′); human 36B4 (NM_001002) forward primer (positions 97–116; 5′-CGACCTGGAAGTCCAACTAC-3′) and reverse primer (positions 205–188; 5′-ATCTGCTGCATCTGCTTG-3′); and human HDAC6 (NM_00436) forward primer (positions 1458–1476; 5′-CAACTGAGACCGTGGAGAG-3′) and reverse primer (positions 1538–1521; 5′-CCTGTGCGAGACTGTAGC-3′); and human PAI1 (NM_000602) forward primer (positions 711–728; 5′-GGCTGGTGCTGGTGAATG-3′) and reverse primer (positions 814–794; 5′-AGTGCTGCCGTCTGATTTGTG-3′); and human HMOX1 (NM_002133) forward primer(positions 1116–1135; 5′-CTCTGGAAAGGAGGAAGGAG-3′) and reverse primer (positions 1277–1258; 5′-TTGAGACAGCTGCCACATTA-3′).

## Additional Information

**How to cite this article**: Deskin, B. *et al*. Requirement of HDAC6 for activation of Notch1 by TGF-β1. *Sci. Rep.* **6**, 31086; doi: 10.1038/srep31086 (2016).
